# Detection of *Mucorales* antigen in bronchoalveolar lavage samples using a newly developed lateral-flow device

**DOI:** 10.1128/jcm.00226-25

**Published:** 2025-06-18

**Authors:** Julie Rousselot, Laurence Millon, Emeline Scherer, Nathalie Bourgeois, Sebastien Imbert, Damien Dupont, Anne Debourgogne, Danièle Maubon, Anne Pauline Bellanger, Christopher R. Thornton

**Affiliations:** 1 Parasitology-Mycology Department, Besançon University Hospital 547597 , Besançon, France; 2 CNRS, Chrono-environnement (UMR 6249), Université Marie et Louis Pasteur 27000 , Besançon, France; 3 MiVEGEC, Parasitology-Mycology, CNRS, IRD, Montpellier University Hospital, University of Montpellier 27037, Montpellier, France; 4 Parasitology-Mycology Department, Bordeaux University Hospital 728253 , Bordeaux, France; 5 Institute of Infectious Agents, Parasitology and Medical Mycology Department, Hospices Civils de Lyon, Croix-Rousse Hospital 684079 , Lyon, France; 6 Parasitology-Mycology Unit, Microbiology Department, Nancy Regional University Hospital-Hôpitaux de Brabois, Vandœuvre-Lès-Nancy, France; 7 Parasitology-Mycology, Grenoble Alpes University Hospital 36724, Grenoble, France; 8 Medical Research Council Centre for Medical Mycology, Faculty of Health and Life Sciences, University of Exeter 3286, Exeter, United Kingdom; University of Calgary, Calgary, Alberta, Canada

**Keywords:** mucormycosis, *Mucorales*, diagnosis, antigen detection, lateral flow device

## Abstract

A murine IgG2b monoclonal antibody, named TG11, binding to an extracellular polysaccharide antigen secreted by all *Mucorales* fungi has been recently developed and integrated into a lateral-flow device (TG11-LFD). The aim of this study was to establish the clinical performance of TG11-LFD on bronchoalveolar lavage (BAL) fluids for the diagnosis of mucormycosis. Thirteen BAL samples from 13 patients with mucormycosis, all of which tested positive for *Mucorales* qPCR (*Mucor/Rhizopus* [*n* = 5], *Lichtheimia* [*n* = 2], *Rhizomucor* [*n* = 5], and *Cunninghamella* [*n* = 1]), were used to assess the TG11-LFD. We also selected 49 BAL samples from 25 patients with other invasive fungal infections (IFI) (aspergillosis, *Pneumocystis* infection, candidiasis, and possible IFI) and from 20 patients without IFI for use as negative controls. The intensities of the test and control lines were recorded using a Cube reader. The diagnostic performance was assessed by analyzing the receiver operating characteristics (ROC) curve with the Jamovi software package (version 2.6.13). The area under the curve of the ROC curve was 0.739. Using a threshold value positivity ≤531 artificial units, the TG11-LFD test has a sensitivity and specificity of 76.92% and 75.51%, respectively, a positive predictive value of 45.45%, and a negative predictive value of 92.5%. In this study, we evaluated the performance of TG11-LFD on clinical samples for the first time and demonstrated its significant potential for enhancing the rapid detection of mucormycosis. Combining antigen detection with qPCR, as successfully applied in the diagnosis of aspergillosis, is likely to yield the most reliable diagnostic approach.

**IMPORTANCE:**

Mucormycosis is a severe emerging, invasive fungal disease caused by fungi in the order *Mucorales*. The mortality rate remains high at approximately 50%. Rapid diagnosis and prompt initiation of targeted treatment are associated with an improved prognosis. Gold standard diagnostic procedures have poor sensitivity and long turnaround times. *Mucorales* polymerase chain reaction in blood and respiratory samples has improved diagnosis, but this technique is not widely available due to high costs and the need for specialist equipment. A prototype lateral-flow device (TG11-LFD) incorporating a mouse monoclonal antibody, which binds to an extracellular polysaccharide antigen specific to *Mucorales* fungi, has been recently developed. In this study, we evaluated for the first time the performance of the TG11-LFD test on clinical bronchoalveolar lavage fluids for diagnosing mucormycosis. With 76.92% sensitivity and 75.51% specificity, this innovative, simple, and affordable approach shows great potential for improving the rapid diagnosis of mucormycosis.

## INTRODUCTION

Mucormycosis is a severe emerging, invasive fungal infection (IFI) caused by fungi in the order *Mucorales*. These are ubiquitous environmental fungi, which can cause rhino-orbital-cerebral, pulmonary, cutaneous, gastro-intestinal, or disseminated infections due to inhalation of airborne spores or via traumatic injuries. The disease is most common in immunocompromised patients with diabetes mellitus or hematological malignancies and in solid organ transplant recipients, but it can also affect immunocompetent individuals, particularly in its cutaneous forms (1–5). The incidence of mucormycosis is increasing worldwide due to the rising prevalence of immunocompromised patients and improvements in diagnosis (6–8). Despite aggressive surgery and antifungal treatment, the all-cause mortality rate remains high at approximately 50% (4, 8, 9). For this reason, the WHO recently assigned *Mucorales* fungi to the “high priority” fungal pathogens group (https://www.who.int/publications/i/item/9789240060241). Rapid diagnosis of *Mucorales* infections and prompt initiation of targeted treatment are associated with improved prognosis (10, 11). At present, diagnosis relies on histopathology and culture of the infecting pathogen from biopsy, but these gold standard procedures have poor sensitivity and long turnaround times. A rapid diagnosis within hours of receipt in a mycology laboratory can be made by direct microscopic observation of broad non-septate hyphae in BAL, but it requires qualified staff and is also an insensitive diagnostic procedure. Diagnosis is improved with the addition of real-time polymerase chain reaction (PCR) in blood and respiratory samples (12–18), but this technique is not widely available due to high cost and the need for specialist equipment. Several groups have designed laboratory-developed tests (LDTs) (13, 15, 19), and commercial kits (MucorGenius by PathoNostics, MycoGENIE Mucorales by Ademtech) are currently available, with similar performances (sensitivity 80%–90%; specificity 90%–100%) when recommendations concerning the pre-analytical variables are respected (20). Antigen tests might enable improved detection of mucormycosis, but the pan-fungal 1-3-β-D-glucan test or *Aspergillus* galactomannan enzyme immunoassay are not suitable for the detection of *Mucorales* fungi as they lack these carbohydrates in their cell walls. Recently, Thornton et al. (21) developed a murine IgG2b monoclonal antibody, named TG11, that is pan-*Mucorales* specific, binding to an extracellular polysaccharide (EPS) antigen secreted by all *Mucorales* fungi. The TG11 antibody has been integrated into a lateral-flow device (TG11-LFD) for rapid detection of the *Mucorales* antigen in human serum or bronchoalveolar lavage (BAL) fluids. In this paper, we investigated for the first time the performance of this LFD test on clinical samples. We retrospectively tested 62 clinical BAL fluids from patients with mucormycosis, other IFI, and no IFI to determine the sensitivity and the specificity of the test.

## MATERIALS AND METHODS

### Sample collection

Sixty-two clinical BAL samples from 58 patients were used to test the TG11-LFD. These samples were collected from patients across six hospitals in France (Besançon, Bordeaux, Grenoble, Lyon, Montpellier, and Nancy) and submitted to the Mycology laboratory, University Hospital of Besançon for *Mucorales*, *Aspergillus,* and/or *Pneumocystis* qPCR testing between 2018 and 2024. The LDT qPCR assays designed by Millon et al. (13) were immediately performed on pellets of fresh samples, and the BAL pellets and some supernatants were stored at −20°C prior to testing with the TG11-LFD.

Across the 62 samples, there were 13 BAL samples from 13 patients classified with “proven or probable mucormycosis” according to the European Organization for Research and Treatment of Cancer/Mycoses Study Group Education and Research Consortium (EORTC/MSGERC) criteria (22), or “mucormycosis PCR-only” or “COVID-associated mucormycosis” as previously described (14, 23). The specific *Mucorales* qPCR, performed at the time of sampling according to the LDT procedure (13, 24), was positive for all patients with the following identification: *Mucor/Rhizopus* (*n* = 5), *Lichtheimia* (*n* = 2), *Rhizomucor* (*n* = 5), and *Cunninghamella* (*n* = 1) (Table 1).

**TABLE 1:** Clinical and biological data and results of TG11-LFD tests of BAL samples from 13 patients with mucormycosis^*a*^

No.	Classification	Co-infection	Sex/ age	Host factors	Clinical form	Antifungal treatment before BAL sampling	Mycology direct examination (sample type)	Mycology culture/ identification (sample type)	Histopathology	Serum/plasma *Mucorales* PCR (Cq) Target	BAL *Mucorales* PCR (Cq) Target	LFD Ag *Mucorales* result
2	Proven mucormycosis	Yes (probable IA)	F/9	AML	Pulmonary + cardiac	None	Negative (BAL)	Negative (BAL)	Positive Panfungal PCR + sequencing (*Rhizopus arrhizus*)	Positive (34) *Mucor/Rhizopus*	Positive (28) *Mucor/Rhizopus*	Positive
3	Proven mucormycosis	No	M/18	ALL	Pulmonary	Caspofungin	Negative (BAL)	Negative (BAL)	Positive *Mucorales* PCR (lung biopsy; Cq = 30; *Rhizomucor*)	Positive (Cq not known) *Rhizomucor*	Positive (28) *Rhizomucor*	Positive
8	Mucormycosis PCR only	No	H/73	AML	Pulmonary	None	Negative (BAL)	Negative (BAL)	Not done	Not done	Positive (34) *Rhizomucor*	Positive
9^*b*^	Mucormycosis PCR only	Yes (probable IA)	H/68	AML HSCT	Pulmonary	Voriconazole then L-AmB	Negative	Negative	Not done	Positive (30) *Mucor/Rhizopus*	Positive (31) *Mucor/Rhizopus*	Negative
10	Mucormycosis PCR only	No	F/17	AML	Disseminated	Voriconazole	Negative (BAL)	Negative (BAL)	Not done	Positive (36;37) *Lichtheimia*	Positive (29) *Lichtheimia*	Positive
12	Mucormycosis PCR only	Yes (*Pneumocystis jirovecii*)	F/43	AML HSCT	Pulmonary	None	Negative (BAL)	Negative (BAL)	Not done	Positive (33) *Rhizomucor*	Positive (33) *Rhizomucor*	Positive
13	Probable mucormycosis	No	M/29	ALL	Pulmonary	None	Positive (BAL)	Positive (BAL): *Cunninghamella* sp.	Negative (BAL)	Positive (29) *Cunninghamella*	Positive (29) *Cunninghamella*	Negative
14	Proven mucormycosis	No	F/23	ALL	Pulmonary	L-AmB	Positive (BAL, lung tissue)	Negative (BAL)	Positive *Mucorales* PCR (lung biopsy; Cq = 29; *Rhizomucor*)	Positive (30) *Rhizomucor*	Positive (35) *Rhizomucor*	Positive
15^*b*^	Proven mucormycosis	No	H/48	CMML	Pulmonary + ORL	L-AmB + isavuconazole	Positive (lung) Negative (sinus)	Positive (sinus: *Rhizopus arrhizus*) not done on lungs and BAL	Positive *Mucorales* PCR (lung biopsy; Cq = 35 *Rhizomucor*) + positive (sinus biopsy; Cq = 25 *Mucor-Rhizopus*)	Positive (33) *Rhizomucor* Positive (36) *Mucor-Rhizopus*	Positive (36) *Rhizomucor*	Negative
16^*b*^	Mucormycosis PCR only	No	H/43	AML	Pulmonary	L-AmB	Negative (BAL)	Negative (BAL)	Not done	Negative	Positive (35) *Mucor/Rhizopus*	Positive
17	Mucormycosis PCR only (COVID-associated Mucormycosis)	Yes (IAPA)	H/75	COVID	Pulmonary	Voriconazole	Not done	Not done	Not done	Negative	Positive (38) *Lichtheimia*	Positive
18	Proven mucormycosis	No	F/48	Cardiac transplant	Cutaneous + pulmonary	L-AmB + isavuconazole	Positive (subcutaneous soft tissue) Negative (BAL)	Positive: *Rhizopus microsporus* (subcutaneous soft tissue) Negative (BAL)	Not done	Positive (39) *Mucor/Rhizopus*	Positive (36) *Mucor/Rhizopus*	Positive
20	Mucormycosis PCR only	No	H/77	AML	Pulmonary	Isavuconazole	Negative (BAL)	Negative (BAL)	Not done	Positive (33) *Mucor/Rhizopus*	Positive (34) *Mucor/Rhizopus*	Positive

^*a*^ Ag, antigen; AML, acute myelogenous leukemia; ALL, acute lymphocytic leukemia; CMML, chronic myelomonocytic leukemia; Cq, quantification cycle; HSCT, hematopoietic stem cell transplant; IA, invasive aspergillosis; IAPA, influenza‐associated pulmonary aspergillosis; and L-AmB, liposomal amphotericin B.

^*b*^ LFD performed on supernatant.

Forty-nine BAL samples from 45 patients without mucormycosis were selected for use as negative controls. We tested a number of different controls: (i) five BAL samples from five patients with probable aspergillosis and two BAL from one patient with chronic aspergillosis, all of which had tested positive for *Aspergillus* using an LDT PCR assay (25); (ii) 13 BAL samples from 13 patients diagnosed with *Pneumocystis* infection, all of which had tested positive for *Pneumocystis* using a commercial PCR kit (*Pneumocystis* ELITe MGB Kit, Ingenius/Elitech); (iii) six BAL samples from five patients with possible IFI (i.e., host factors and radiological signs suggestive of IFI, but no mycological criteria); (iv) one BAL sample from one patient with *Candida* endocarditis; (v) three BAL samples from three patients without IFI but with positive mycological qPCR or culture (one positive *Aspergillus* qPCR, two positive culture [*Candida albicans* and *Candida glabrata*]); (vi) 19 BAL samples from 17 patients without IFI, all negative by fungal qPCR and mycological culture. All 49 BAL samples used as negative controls were negative for *Mucorales* PCR.

Biological material was obtained only for standard diagnosis on the basis of the physicians’ prescriptions. Clinical data were collected retrospectively from medical records and pseudonymized for the analysis.

### TG11-LFD tests with clinical BAL samples

**Fig 1:**
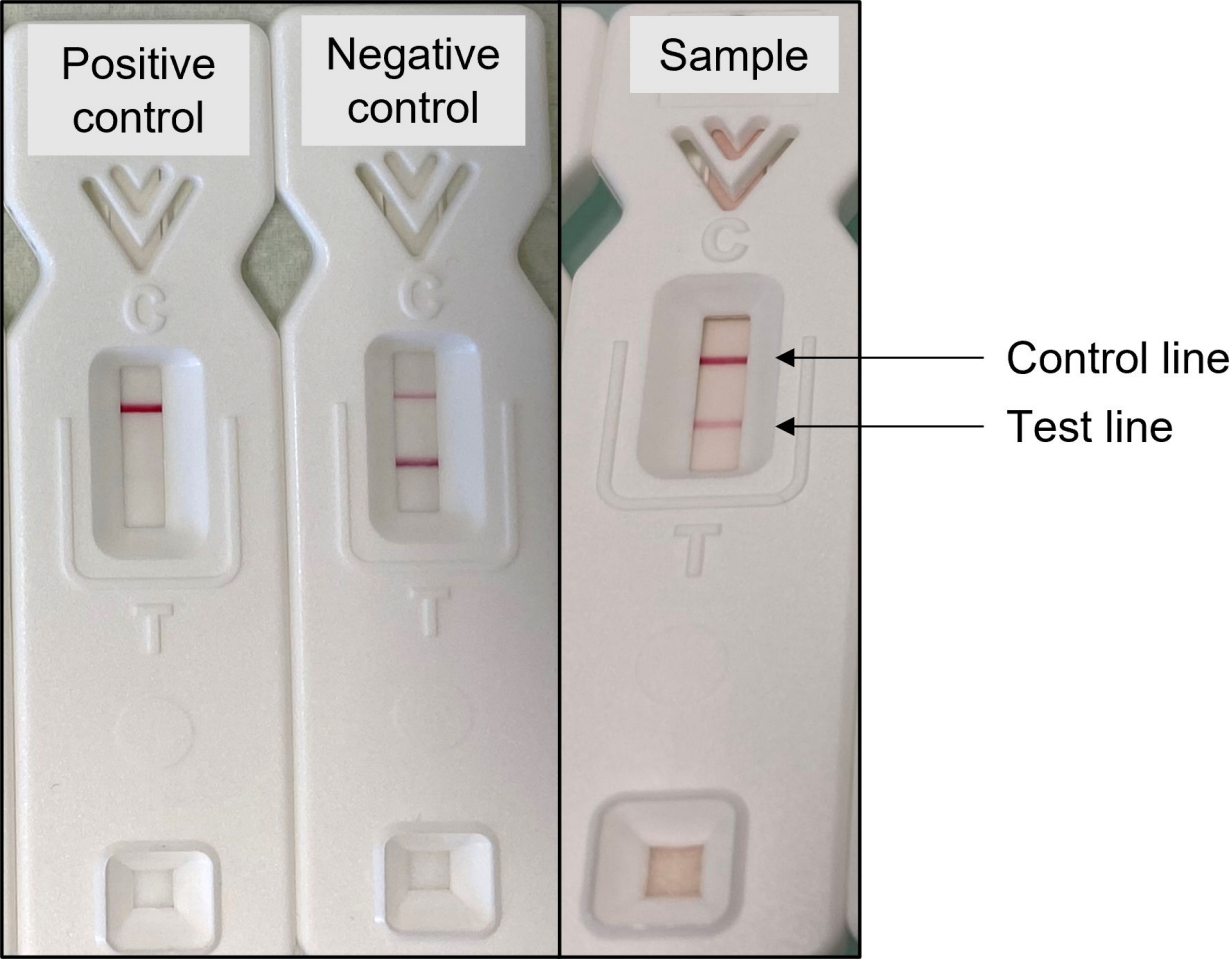
Illustrative results of competitive TG11-LFD tests showing reduction of test (T) line intensities with positive control (SRB with purified EPS antigen) and with BAL sample 2, and maintenance of T line with the negative control (SRB only).

TG11-LFD tests were performed on the 62 BAL samples selected for this study. After thawing, 20 µL of BAL pellet or supernatant (depending on the samples available) was mixed with 80 µL of serum running buffer (SRB) (21), and the resulting 100 µL was added to the TG11-LFD. One hundred microliters of SRB alone was used as the negative control. The tests were performed on 29 BAL supernatants and 33 BAL pellets (Table 2). The positive control comprised purified EPS from *Rhizopus arrhizus* var. *arrhizus* (21) and was prepared by mixing 10 µL of the positive control and 90 µL of SRB, and 100 µL was added to the TG11-LFD test. The intensities of the test (T) and control (C) lines were recorded after 30 min as artificial units (a.u.) using a Cube reader (21). The TG11-LFD is a competitive test; thus, a positive result corresponds to a low intensity of the test line (Fig. 1).

**TABLE 2:** Clinical classification and results of TG11-LFD tests (raw data and interpretation) for the 62 BAL samples tested^*a*^

Sample^*b*^^,*c*^	Underlying disease	Clinical diagnosis	Antifungal treatment before BAL sampling	Results of BAL culture	Results of BAL PCR	Test line value of LFD (a.u.)^*d*^	Interpretation with threshold value of ≤531 a.u. (T line)
2a	AML	MM PCR only	None	Negative	*Mucor/Rhizopus*	218.9	Positive
3a	ALL	Proven MM	Caspofungin	Negative	*Rhizomucor*	218.5	Positive
8a	AML	MM PCR only	None	*Staphylococcus haemolyticus* and *Candida kefyr*	*Rhizomucor*	494.5	Positive
9b	AML/HSCT	MM PCR only + probable IA	Voriconazole then L-AmB	Negative	*Mucor/Rhizopus*	748.1	Negative
10a	AML	MM PCR only	Voriconazole	Negative	*Lichtheimia*	442.3	Positive
12a	AML/HSCT	MM PCR only	None	Negative	*Rhizomucor*	6.6	Positive
13a	ALL	Probable MM	None	Negative	*Cunninghamella*	838.9	Negative
14a	ALL	Proven MM	L-AmB	*Enterococcus faecium*	*Rhizomucor*	366	Positive
15b	CMML	Proven MM	L-AmB + isavuconazole	Negative	*Rhizomucor*	826.4	Negative
16b	AML	MM PCR only	L-AmB	Negative	*Mucor/Rhizopus*	531	Positive
17a	COVID	COVID-associated MM + IAPA	Voriconazole	Unknown	*Lichtheimia/* *Aspergillus*	296.5	Positive
18a	Cardiac transplant	Proven MM	L-AmB + isavuconazole	Yeasts	*Mucor/Rhizopus*	204	Positive
20a	AML	MM PCR only	Isavuconazole	*Staphylococcus aureus* and *Enterococcus faecium*	*Mucor/Rhizopus*	472	Positive
22b	Lung transplant	Probable IA	None	*Stenotrophomonas maltophilia*	*Aspergillus*	428.5	Positive
23b	Pulmonary embolism	Probable IA	None	Negative	*Aspergillus*	828.4	Negative
24a	*Mycobacterium* infection	Chronic IA	Unknown	*Aspergillus fumigatus*	*Aspergillus*	752.5	Negative
27b (same patient as 24)	*Mycobacterium* infection	Chronic IA	Unknown	Negative	*Aspergillus*	832	Negative
25a	Sarcoidosis	Probable IA	None	*Candida lusitaniae*	*Aspergillus* and *Legionella pneumophila*	228	Positive
26a	COPD/influenza	Probable IA	None	*Stenotrophomonas maltophilia* and *Candida tropicalis*	*Aspergillus*	509.5	Positive
28b	Chronic bronchitis/influenza/bacterial superinfection	No IFI	None	Negative	*Aspergillus*	327	Positive
29b	Alcoholism/influenza	IAPA	None	*Pseudomonas aeruginosa* and *Candida albicans*	*Aspergillus*	758.9	Negative
30b	Cirrhosis	*P. jirovecii* infection	None	*Staphylococcus aureus*	*Pneumocystis*	675.5	Negative
31b	Hodgkin lymphoma	*P. jirovecii* infection	None	Negative	*Pneumocystis*	838.5	Negative
32b	Renal transplant	*P. jirovecii* infection	None	*Haemophilus influenzae*	*Pneumocystis*	671.5	Negative
33b	Lymphoma	*P. jirovecii* infection	None	Not done	*Pneumocystis*	552.5	Negative
34b	Renal transplant	*P. jirovecii* infection	None	Negative	*Pneumocystis*	830.4	Negative
35b	Lymphoma	*P. jirovecii* infection	None	Negative	*Pneumocystis*	564.1	Negative
36a	Sarcoidosis	*P. jirovecii* infection	Unknown	Negative	*Pneumocystis*	760.4	Negative
37b	Lymphoma	*P. jirovecii* infection	None	Negative	*Pneumocystis*	688.4	Negative
38b	Lung cancer	*P. jirovecii* infection	Unknown	Negative	*Pneumocystis*	750.2	Negative
39b	ALL	*P. jirovecii* infection	Unknown	*Candida albicans*	*Pneumocystis*	838.4	Negative
40b	Lymphoma	*P. jirovecii* infection	Unknown	Negative	*Pneumocystis*	838	Negative
42b	Myelodysplasia	*P. jirovecii* infection	Isavuconazole	*Escherichia coli*	*Pneumocystis*	542.5	Negative
43b	Rheumatoid arthritis	*P. jirovecii* infection	Unknown	*Pseudomonas aeruginosa, Candida kefyr,* and *Penicillium* sp.	*Pneumocystis*	828.7	Negative
44b	ALL	Possible IFI	L-AmB	*Candida dubliniensis*		183	Positive
45a	Lung cancer/ rheumatoid arthritis	No IFI	Unknown	*Candida albicans*		539.5	Negative
46b	Obstructive ventilatory disorder	No IFI	None	*Klebsiella aerogenes, Proteus vulgaris* group*, Pseudomonas aeruginosa, Candida albicans,* and *Candida glabrata*		539.5	Negative
47a	Unknown	No IFI	Unknown	Not done	None	760.8	Negative
48a	Unknown	No IFI	Unknown	Not done	None	835	Negative
49a	Unknown	No IFI	Unknown	Not done	None	679.4	Negative
50b	Medullar aplasia	Possible IFI	L-AmB	Negative	None	506	Positive
51b	AML	Possible IFI	Isavuconazole	Negative	None	455	Positive
52a	Lymphoma	No IFI	L-AmB	Negative	None	560.1	Negative
53b	Cardiac disease	No IFI	None	Negative	None	419.4	Positive
57b (same patient as 53)	Cardiac disease	No IFI	None	Negative	None	362.9	Positive
54a	Unknown	No IFI	Unknown	Not done	None	547	Negative
55a	Acute respiratory failure	No IFI	None	*Aspergillus ochraceus*	None	760.5	Negative
56b	COPD	No IFI	None	*Corynebacterium striatum*	None	830	Negative
59b (same patient as 56)	COPD	No IFI	None	*Corynebacterium striatum* and *Candida albicans*	None	537.5	Negative
58a	COPD/*Klebsiella* pneumonitis	No IFI	None	*Klebsiella pneumoniae*	None	548.5	Negative
60b	CMML	Possible IFI	Unknown	Negative	None	394.5	Positive
61a	Unknown	No IFI	Unknown	Not done	None	477.4	Positive
62a	Unknown	No IFI	Unknown	Not done	None	835	Negative
63a	Unknown	No IFI	Unknown	Not done	None	765.5	Negative
64a	Unknown	No IFI	None	Not done	None	689.5	Negative
65b	Asthma/respiratory failure	No IFI	Unknown	*Candida albicans*	None	680	Negative
66a	Unknown	No IFI	Unknown	Not done	None	836	Negative
67b	Lymphoma/liver transplant	Possible IFI	None	Negative	None	760	Negative
71b (same patient as 67)	Lymphoma/liver transplant	Possible IFI	None	Negative	None	191.9	Positive
68a	Unknown	No IFI	None	Negative	None	834	Negative
69a	Lung and breast cancer	Endocarditis *Candida albicans*	Caspofungin	Negative	None	537	Negative
70a	Myocardial infarction	No IFI	None	*Pseudomonas aeruginosa* and *Candida albicans*	None	834	Negative

^*a*^ AML, acute myelogenous leukemia; ALL, acute lymphocytic leukemia; CMML, chronic myelomonocytic leukemia; COPD, chronic obstructive pulmonary disease; HSCT, hematopoietic stem-cell transplantation; IA, invasive aspergillosis; IAPA, influenza-associated pulmonary aspergillosis; L-AmB, liposomal amphotericin B; and MM, mucormycosis.

^*b*^ Lower case “a” indicates that LFD performed on BAL pellet.

^*c*^ Lower case “b” indicates that LFD performed on BAL supernatant.

^*d*^ The internal control (C) line value for all LFD tests was ≥382 a.u.

### Statistical analysis

The diagnostic performance of the TG11-LFD test was assessed by analyzing the receiver operating characteristics (ROC) curve with the Jamovi software package (version 2.6.13) in accordance with other studies determining the diagnostic performance of LFD tests from test (T) line intensities in artificial units, derived from Cube reader outputs (26). The ROC curve analyses were performed by assigning samples to two groups: samples from patients with mucormycosis (*n* = 13) versus samples from patients without mucormycosis (samples from patients with other IFI [aspergillosis or *Pneumocystis* infection] and patients without IFI [*n* = 49]). The statistical analyses were performed using the a.u. values of the TG11-LFD test (T) line. The threshold that best distinguished the positive samples from the negative samples was determined with the most effective combination of sensitivity and specificity.

## RESULTS

**Fig 2:**
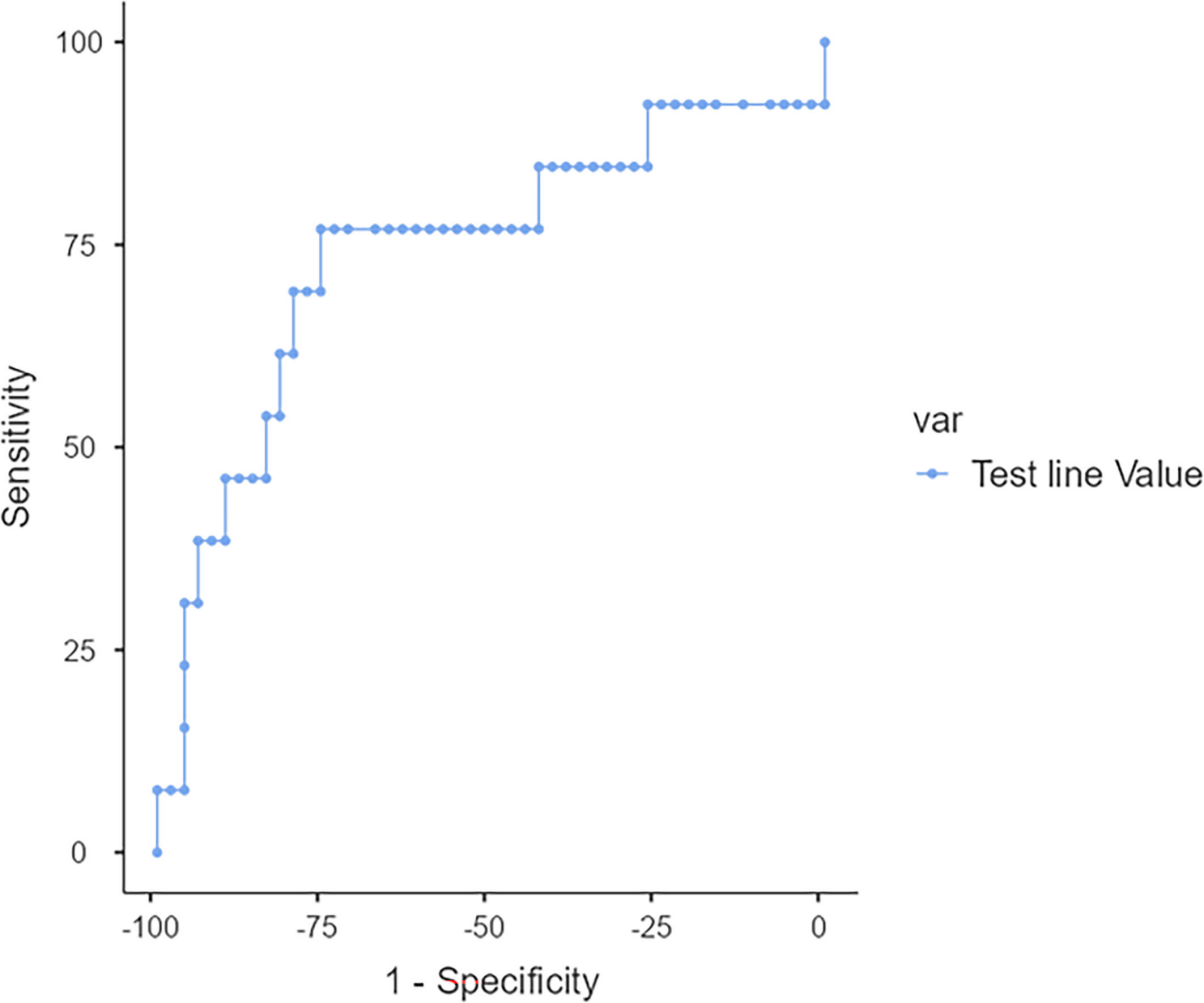
ROC curve analysis for test positivity (Jamovi software analysis). The ROC curve was generated using artificial unit values of the test (T) line to distinguish samples from patients with mucormycosis (*n* = 13) and without mucormycosis (*n* = 49). The AUC is 0.739, and the optimal threshold for test positivity was determined as ≤531 a.u., with T line a.u. values below this indicating a positive LFD result.

The area under the curve (AUC) of the ROC curve was 0.739 (Fig. 2). ROC analyses showed that the most appropriate test (T) line a.u. value for LFD positivity is ≤531 a.u. Consequently, a T line value below this threshold value (531 a.u.) indicated a positive test result for the *Mucorales* EPS antigen. Using this threshold value, the results were as follows: 10 true-positive, 37 true-negative, 12 false-positive, and 3 false-negative samples (Table 2). Consequently, the sensitivity of the TG11-LFD test was found to be 76.92%, specificity was 75.51%, with a positive predictive value of 45.45% and a negative predictive value of 92.5%.

The three false-negative samples correspond to infection with three different *Mucorales* genera identified by qPCR assays, namely *Mucor/Rhizopus*, *Rhizomucor,* and *Cunninghamella* (Table 1). All three patients had positive *Mucorales* qPCR on serum prior to BAL sampling, and two of them had received liposomal amphotericin B (L-Amb) for 2–4 days at the time of BAL.

The 12 false-positive results were observed for 11 patients: 3 patients without IFI (including one with a positive *Aspergillus* PCR on BAL, and one with 2 consecutive samples (53 and 57) positive for TG11-LFD), 3 patients with probable invasive aspergillosis, and 5 patients with possible IFI (Table 2). Among the five false positives observed in patients with possible IFI, one patient underwent two BAL samplings at a 10 day interval (samples 67 and 71), and imaging (thoracic computed tomography) was performed at the time of each BAL sampling. Lesions suggestive of fungal infection (“condensation of infectious origin”) were reported at the time of the first BAL sampling (negative by TG11-LFD). Despite negative bacteriological results, the patient received antibiotics (meropenem followed by tazocilline). An increase in lung lesions was reported 10 days later, at the time of the second BAL sampling (positive by TG11-LFD). The patient died 3 days later without any antifungal treatment. Similarly, for two other patients with possible IFI (samples 44 and 60), imaging reports were suggestive of fungal infection, but they did not receive any antifungal treatment, and they died 10 and 20 days later, respectively. For the remaining two patients, in the context of the suggestive imaging and the absence of mycological criteria for aspergillosis, a mold-active antifungal treatment was initiated (L-Amb, then isavuconazole for one; isavuconazole only for the other). Both patients presented a favorable outcome and were alive at D90. The percentage of false positives was 31% (6/16) for hematology/oncology patients and 21% (6/29) for non-hematology/oncology patients.

We examined microbiological results for BAL samples with false-positive results (Table 2). There was a single sample with a positive culture of *Stenotrophomonas maltophilia*, one sample with a positive culture of *Stenotrophomonas maltophilia* and *Candida tropicalis,* two samples with a positive yeast culture (*Candida lusitaniae* and *Candida dubliniensis*), and six samples for which the microbiological cultures were negative. Microbiological results were not available for the remaining two samples. The two BAL samples with positive *Stenotrophomonas maltophilia* culture were also positive for *Aspergillu*s PCR.

## DISCUSSION

In this study, we evaluated a newly developed prototype LFD test incorporating a mouse IgG2b monoclonal antibody, code name TG11, which binds to an extracellular polysaccharide antigen specific to *Mucorales* fungi (21). This retrospective evaluation was performed with 62 BAL samples collected from 13 patients with mucormycosis, from 25 patients with other IFI (probable or chronic aspergillosis, *Pneumocystis* infection, invasive candidiasis, and possible IFI) and from 20 patients without IFI. Under these conditions, we have shown that the TG11-LFD test has a sensitivity of 76.92% and a specificity of 75.51%.

Invasive mucormycosis is a highly aggressive angio-invasive infection, especially in immunocompromised patients. *Mucorales* fungi are well established as the second most common mold pathogens after *Aspergillus*, but it was the dramatic increase in cases of mucormycosis during the coronavirus pandemic that exposed serious weaknesses in diagnostic capability, which continues to rely on insensitive and laborious culture of *Mucorales* fungi from tissue biopsies as a gold standard for detection (27). Although *Mucorales* qPCR on serum and BAL has greatly improved the diagnostic accuracy for mucormycosis, with sensitivity of 80%–85% and specificity of 90%–99% for some LDT and commercial assays in recent studies (14, 16, 17), it is not universally available, especially in low- to middle-income countries. Consequently, the development of a rapid antigen test, such as the lateral-flow device evaluated here, provides a unique opportunity for access to diagnostics that meet the ASSURED (affordable, sensitive, specific, user-friendly, rapid, equipment-free, delivered) criteria for the developing world (28). Even though the TG11-LFD test is less sensitive and specific than qPCR, the ease with which it can be performed and the speed with which results can be obtained make it a very attractive tool for diagnosing pulmonary mucormycosis. Moreover, its sensitivity is superior to culture, where over 50% of samples fail to produce viable propagules, even when fungal elements are visible in histology (29).

Three false-negative results were obtained in three patients, who had a positive *Mucorales* qPCR on the same BAL sample. One patient was classified as “mucormycosis PCR only” but presented several positive qPCR results on serum for the same target organisms (*Mucor*/*Rhizopus*), thereby ensuring the diagnosis of mucormycosis. The second patient had probable mucormycosis with a positive culture of a *Cunninghamella* species. The third patient had proven mucormycosis, with the qPCR identification of *Rhizomucor* in lung tissue and culture of *Rhizopus arrhizus* from the sinus. These false-negative TG11-LFD results might have been due to BAL storage. However, the TG11-LFD test was conducted using BAL pellets stored at −20°C from 2019, 2021, and 2022 for these three patients, respectively, and the period of BAL storage was similar for all other patients classified with mucormycosis (from 2018 to 2024). It is also possible that treatment with L-Amb initiated 2 or 4 days before BAL sampling affected antigen detection in two of the three patients.

False-positive LFD results were observed for 11 patients. Of note, 5 out of the 11 patients were classified with possible IFI according to the EORTC/MSGERC criteria. Indeed, they presented risk factors for invasive fungal (hematological malignancies and liver transplant) and pulmonary radiological signs consistent with a fungal infection. These cases may, therefore, not represent true false positives, but rather undetected mucormycosis that other mycological tools failed to identify. The negative qPCR results could be related to infective *Mucorales* species not targeted by the specific LDT assay used or technical variability of nucleic acid extraction from BAL samples (30). Similarly, three patients with probable IA gave false positives with the TG11-LFD test. Despite negative *Mucorales* qPCR on the same BAL samples, a mixed *Aspergillu*s-*Mucorales* infection cannot be excluded, as mixed infections now represent ~25% of invasive mold infections in immunosuppressed patients (8). Of these three patients, two also had a *Stenotrophomonas maltophilia* BAL positive culture. Two additional false-positive tests were observed in patients with a BAL positive culture for yeast. However, it is not possible to conclude about potential cross-reactivity because of the low number of samples. Finally, four patients without radiological criteria for IFI, including one with cardiac disease and one with chronic bronchitis and influenza as underlying disease, had false-positive results.

This study has some limitations. The TG11-LFD tests were performed retrospectively with frozen BAL, and repeated freeze-thawing could have had an impact on antigen detectability. Moreover, for some samples, tests were performed mainly on BAL pellet (*n* = 33) and not BAL supernatant, and this might also influence test outcome. The threshold value was determined using this initial series of clinical samples and will need to be validated with an independent series, which may also help to define a range around the threshold. A prospective study, with a larger sample size, standardized sampling and storage conditions, and prospective collection of clinical data, is necessary to confirm our preliminary findings. Performance under immunosuppressive conditions will be an important point to consider. Furthermore, the monoclonal antibody TG11 was tested for reactivity with antigens from *in vitro* cultures of a wide range of mucoralean molds and for cross-reactivity with non-mucoralean yeasts and molds of clinical importance, but not with other microorganisms that can be found in BAL samples such as respiratory bacteria. Further experimental and clinical evaluations are necessary to better assess cross-reactivity with other respiratory pathogens. Nevertheless, this first study with clinical samples showed that the TG11-LFD is a very promising tool with sensitivity and specificity of up to 75%. The performance of the test might be even better since positive results were also observed in five patients with possible IFI, three of them with rapid fatal evolution without antifungal treatment, and two of them with a favorable outcome under *Mucorales*-active antifungal treatment. These five patients might, therefore, have had mucormycosis missed during the initial diagnosis.

This innovative tool holds significant potential for improving the rapid detection of mucormycosis, particularly in low- to middle-income countries where access to sophisticated diagnostic platforms is limited. It might also help to support *Mucorales* qPCR, providing distinct but complementary diagnostic capabilities, particularly where species not targeted by specific qPCR assays are present. Combining multiple biomarkers, cell-free DNA detection, and antigen detection, as demonstrated in the case of *Aspergillus* qPCR and galactomannan for aspergillosis, is likely to yield the most reliable diagnostic approach (31) since antigen and DNA release are known to occur at different times during the progression of IFI. Nevertheless, evaluating the performance of *Mucorales* TG11-LFD on serum samples is an important next step, as it might enable non-invasive detection of circulating EPS antigen without the need for patients to undergo invasive BAL procedures. The TG11-LFD could also be evaluated with alternative biofluids such as urine.
